# Supplementary material for: Reducing aggregation caused quenching effect through co-assembly of PAH chromophores and molecular barriers
Source: Nat Commun. 2019 Jan 11;10:169. doi: 10.1038/s41467-018-08092-y (PMC6329816; doi:10.1038/s41467-018-08092-y)
Supplement: Supplementary file 1 — Supplementary Information [file 41467_2018_8092_MOESM1_ESM.pdf]

Reducing Aggregation Caused Quenching Effect through Co-Assembly of PAH Chromophores and Molecular Barriers

Supporting Information

Huang et al.

Reducing Aggregation Caused Quenching Effect through Co-Assembly of PAH Chromophores and Molecular Barriers

Supporting Information

Yinjuan Huang[1], Jie Xing[2], Qiuyu Gong[3], Li-Chuan Chen[4], Guangfeng Liu[1], Changjiang Yao[1], Zongrui Wang[1], Hao-Li Zhang[4*], Zhong Chen[1], Qichun Zhang[1]*

[1]School of Materials Science and Engineering, Nanyang Technological University (Singapore), 639798, Singapore; [2]Ningbo Institute of Materials Technology and Engineering, Chinese Academy of Sciences, No. 1219 ZhongGuan West Road, 315201, Ningbo, China; [3]Department of Chemistry, National University of Singapore, 3 Science Drive, Singapore, 117543, Singapore; [4]State Key Laboratory of Applied Organic Chemistry, Lanzhou University, Tianshui Southern Road 222, Lanzhou, Gansu Province, P. R. China.

E-mail: qczhang@pmail.ntu.edu.sg; Haoli.zhang@lzu.edu.cn

Contents

Supplementary method. Preparation of micro/nano co-crystals.

Supplementary Table 1. PLQYs and the corresponding enhancements of the co-crystals.

Supplementary Figure 1. The curves of the PLQYs versus the mole ratios of PAHs to OFN.

Supplementary Figure 2. FTIR spectra of OFN crystals (blue line), Per MS (black line) and Per/OFN MW (red line).

Supplementary Figure 3. FTIR spectra of OFN crystals (blue line), Cor MW (black line), and Cor/OFN MW (red line).

Supplementary Figure 4. Raman spectra of Per MS (black line) and Per/OFN MW (red line).

Supplementary Figure 5. Raman spectra of Cor MW (black line) and Cor/OFN MW (red line).

Supplementary Figure 6. DSC curves of OFN crystals (blue line), Per MS (black line) and Per/OFN MW (red line).

Supplementary Figure 7. DSC curves of OFN crystals (blue line), Cor MW (black line) and Cor/OFN MW (red line).
Supplementary Figure 8. Experimental XRD spectra and calculated PXRD for Per/OFN.
Supplementary Figure 9. Experimental XRD spectra and calculated PXRD for Cor/OFN.
Supplementary Figure 10. Packing structure of Per and OFN.
Supplementary Figure 11. The distances between two PAH planes in Per/OFN cocrystals.
Supplementary Figure 12. Packing structure of Cor and OFN.
Supplementary Figure 13. The distances between two PAH planes in Cor/OFN cocrystals.
Supplementary Table 2. Crystallographic data and structure refinement parameters of Ant-OFN co-crystals and Cor-OFN co-crystals.
Supplementary Figure 14. SEM image of OFN crystals.
Supplementary Figure 15. SEM and AFM images of Per MS and Per/OFN MW.
Supplementary Table 3. Calculated attachment energies of different crystal facets of Per/OFN co-crystals.
Supplementary Figure 16. TEM images and selected area electron diffraction (SAED) of Per/OFN MW.
Supplementary Figure 17. SEM and AFM images of Cor MW and Cor /OFN MW.
Supplementary Table 4. Calculated attachment energies of different crystal facets of Cor/OFN co-crystal.
Supplementary Figure 18. TEM images and SAED of Cor/OFN MW.
Supplementary Figure 19. Schematic of interactions between OFN and PAHs.
Supplementary Figure 20. Solid UV-vis and PL spectra of THF solution of Per and Per MS powder.
Supplementary Figure 21. UV-vis and PL spectra of THF solution of Cor and Cor MW powder.
Supplementary Table 5. A_i and T_i values of Per MS, Per/OFN MW, Cor MW and Cor/OFN MW.
Supplementary Figure 22. The pattern process of "NTU".
Supplementary Figure 23. AFM images of nano sheets of Per NS and Per /OFN NP.
Supplementary Figure 24. AFM images of nano rods of Cor NR and Cor/OFN NR.

Supplementary Figure 25. Dispersion photos of nano cocrystals after being aged for 6 months.
Supplementary Figure 26. AFM images of the nano cocrystals after being aged for 6 months.
Supplementary Figure 27. UV-vis and PL spectra of the water dispersions for Per NS and Per/OFN NP.
Supplementary Figure 28. UV-vis and PL spectra of the water dispersions for Cor NR and Cor/OFN NR.
Supplementary Figure 29. PL spectra of the water dispersions for the nano cocrystals after 6 months placement.
Supplementary Figure 30. Cytotoxicity tests.
Supplementary Reference

Supplementary method

Preparation of micro/nano co-crystals. In this work, nano co-assembly method was used to prepare the micro/nano co-crystals. Coronene (Cor), perylene (Per), pyrene (Pyr) and anthracene (Ant), which have strong π-π stacking and present aggregation caused quench (ACQ) in solid state[1-4], were chosen to study how to reduce the ACQ Effect of chromophores.

Supplementary Table 1. PLQYs and the corresponding enhancements of the co-crystals.

Chromophore/OFN ratio		1:0 (Raw)[a]	1:0	1:0.5	1:1	1:2	1:5	1:8	1:10
Per/OFN MW	PLQY(%)	4.6 (8.3) [c]	7.9	9.8	16.3	15.2	14.9	13.2	14.8
	Enhancement(%)[b]	-	72	113	254	230	224	187	222
Cor/OFN MW	PLQY(%)	3.4 (4.6) [c]	5.8	8.3	11.4	10.7	9.7	10.9	10.4
	Enhancement(%)[b]	-	70	144	235	206	185	220	205
Ant/OFN MP	PLQY(%)	27.7	54.6		48.9	-	-	-	-
	Enhancement(%)[b]	-	97		77	-	-	-	-
Pyr/OFN MP	PLQY(%)	18.6	22.0		20.5	-	-	-	-
	Enhancement(%)[b]	-	18		10	-	-	-	-

[a] The PLQYs for raw solid chromophores as received, the value in the parenthesis are the PLQY of the solutions of corresponding PAHs in $CHCl_3$; [b] enhancement of the co-crystals compared to raw solid chromophores. The PLQYs are the average values of three tests. [c] The PLQYs of PAHs in solid (the value outside the parentheses) and chloroform solution (the value in parenthesis).

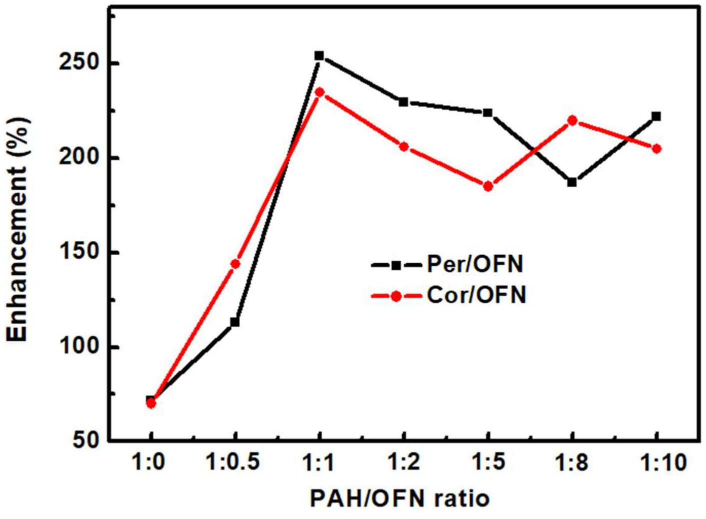

Supplementary Figure 1. The curves of the PLQYs versus the mole ratios of PAHs to OFN. The curves of the PLQYs versus the mole ratios of PAHs to OFN for micro Per/OFN (black line) and Cor/OFN (red line).

Concluded from above photoluminescence quantum yield (PLQY) results presented in Supplementary Table 1, the samples for Per/OFN and Cor/OFN showed the highest PLQY under the PAH/OFN ratio of 1:1, which were chosen together with the control ratio (1:0) to be used in further experiments. The micro samples for Per/OFN 1:0, Per/OFN 1:1, Cor/OFN 1:0 and Cor/OFN 1:1 were named as Per MS, Per/OFN MW, Cor MW and Cor/OFN MW, respectively.

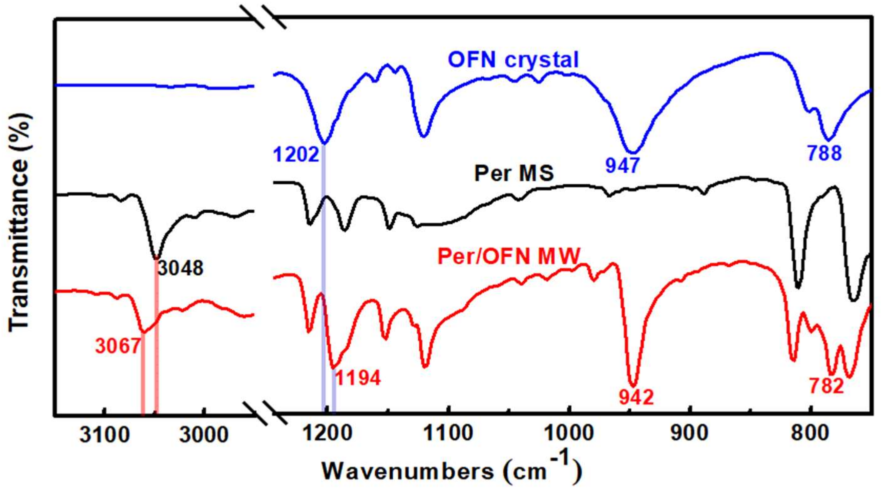

Supplementary Figure 2. FTIR spectra of OFN crystals (blue line), Per MS (black line) and Per/OFN MW (red line). The appearance of the characteristic peaks of C-F bond at 782 cm^{-1} and 942 cm^{-1} (the stretching of C-F in OFN [5]) in the spectra of Per/OFN MW demonstrated the successful doping of OFN molecule into Per to form Per/OFN co-crystals. Moreover, because of being surrounded by electron rich Per, the stretching peaks of OFN at 1202 cm^{-1} shifted to 1194 cm^{-1}; conversely, the strong absorption band at 3048 cm^{-1} (the stretching of Ar-H in Per) of Per shifted to 3067 cm^{-1} after doping electron-deficient OFN, which further conformed the succeful packing between electron rich Per and electron-deficient OFN.[6]

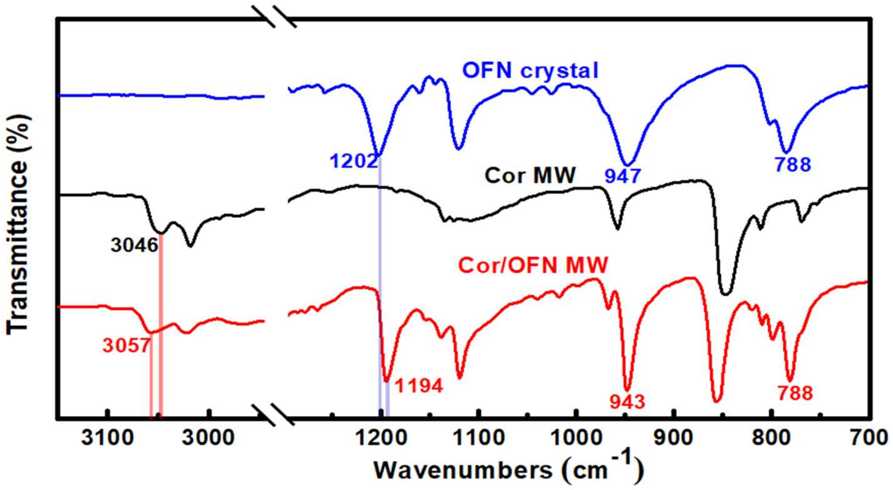

Supplementary Figure 3. FTIR spectra of OFN crystals (blue line), Cor MW (black line), and Cor/OFN MW (red line). The appearance of the characteristic peaks at 780 cm^{-1} and 943 cm^{-1} (the stretching of C-F in OFN) from C-F bond in the spectra of Cor/OFN MW demonstrated the successful doping of OFN molecule into Cor to form Cor/OFN MW. In addition, the stretching peaks of OFN at 1202 cm^{-1} shifted to 1194 cm^{-1} after packing with electron rich Cor, and the strong absorption band at 3046 cm^{-1} (the stretching of Ar-H in Cor) of Cor shifted to 3057 cm^{-1} after doping electron-deficient OFN, which further conformed the succeful preparation of Cor/OFN Co-crystals.

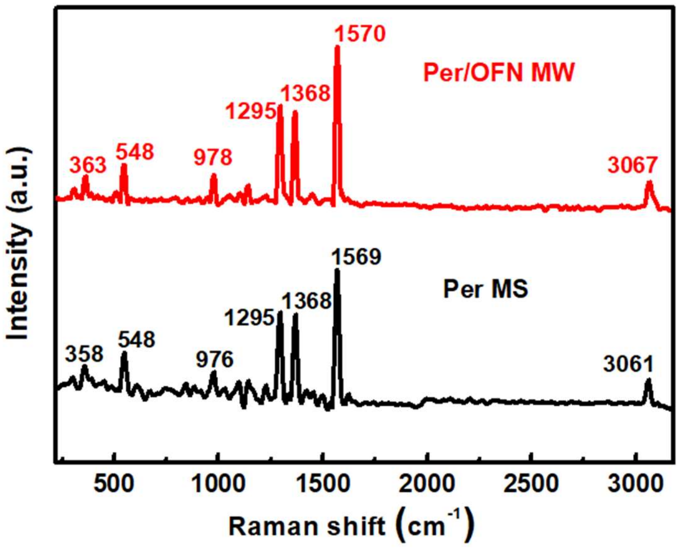

Supplementary Figure 4. Raman spectra of Per MS (black line) and Per/OFN MW (red line). The peaks at 358 cm^{-1}, 976 cm^{-1} and 3061 cm^{-1} from Per MS shifted to 363 cm^{-1}, 978 cm^{-1} and 3067 cm^{-1}, respectively, which indicate the decreased electron density of Per in Per/OFN MW.[6]

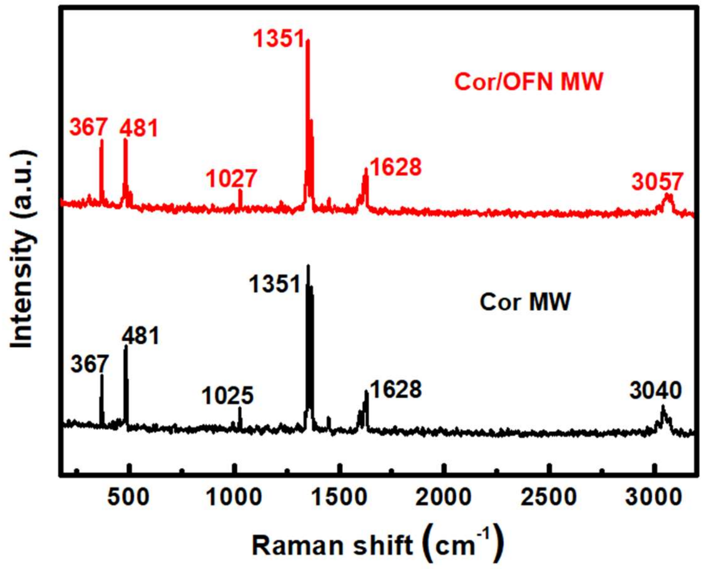

Supplementary Figure 5. Raman spectra of Cor MW (black line) and Cor/OFN MW (red line). The peaks at 1025 cm^{-1} and 3040 cm^{-1} from Cor MW shifted to 1027 cm^{-1} and 3057 cm^{-1}, respectively, which indicate the decreased electron density of Cor in Cor/OFN MW.[6]

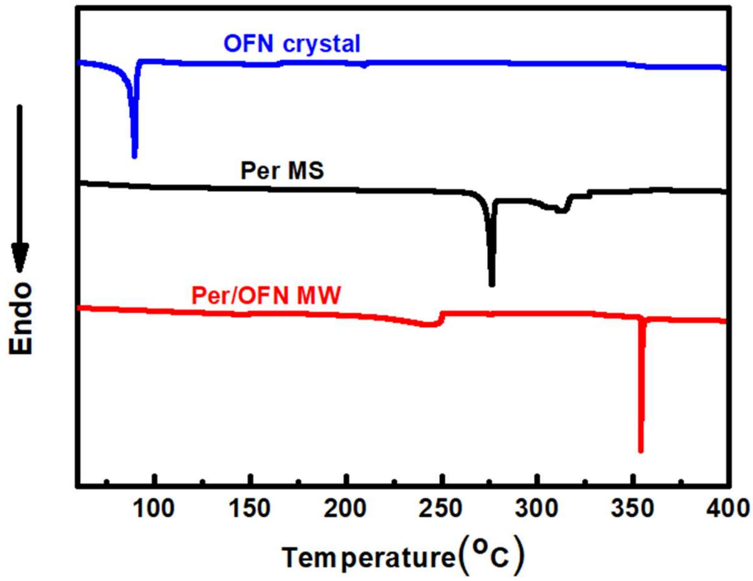

Supplementary Figure 6. DSC curves of OFN crystals (blue line), Per MS (black line) and Per/OFN MW (red line). The temperature at the single sharp peaks displayed the melting points. Evidently, after being doped OFN, the melting point of Per/OFN MW increased from 252°C to 355°C.

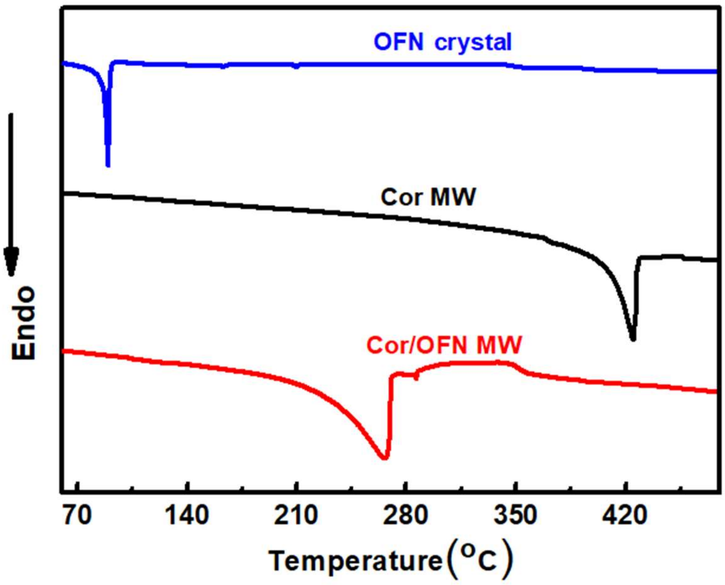

Supplementary Figure 7. DSC curves of OFN crystals (blue line), Cor MW (black line) and Cor/OFN MW (red line). For Cor MW and Cor/OFN MW, they have already decomposed (indicated by the much broader peaks) before the melting points appearred due to the unstability and high melting point of Cor.

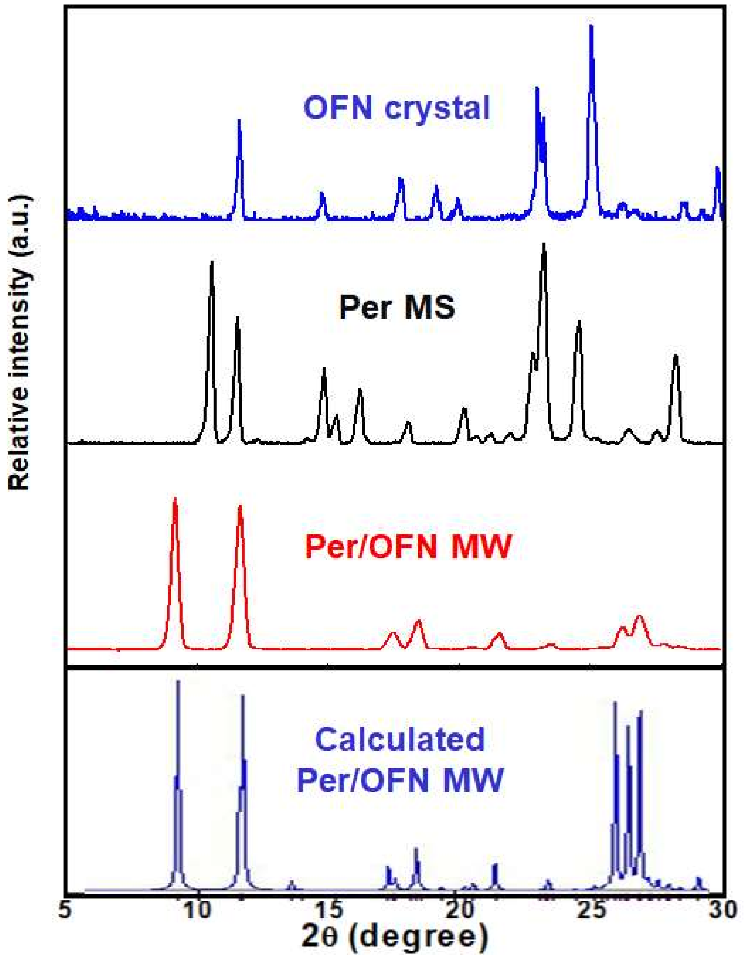

Supplementary Figure 8. Experimental XRD spectra and calculated PXRD for Per/OFN. Experimental XRD spectra of OFN crystals, Per MS and Per/OFN MW and calculated PXRD of Per/OFN MW.

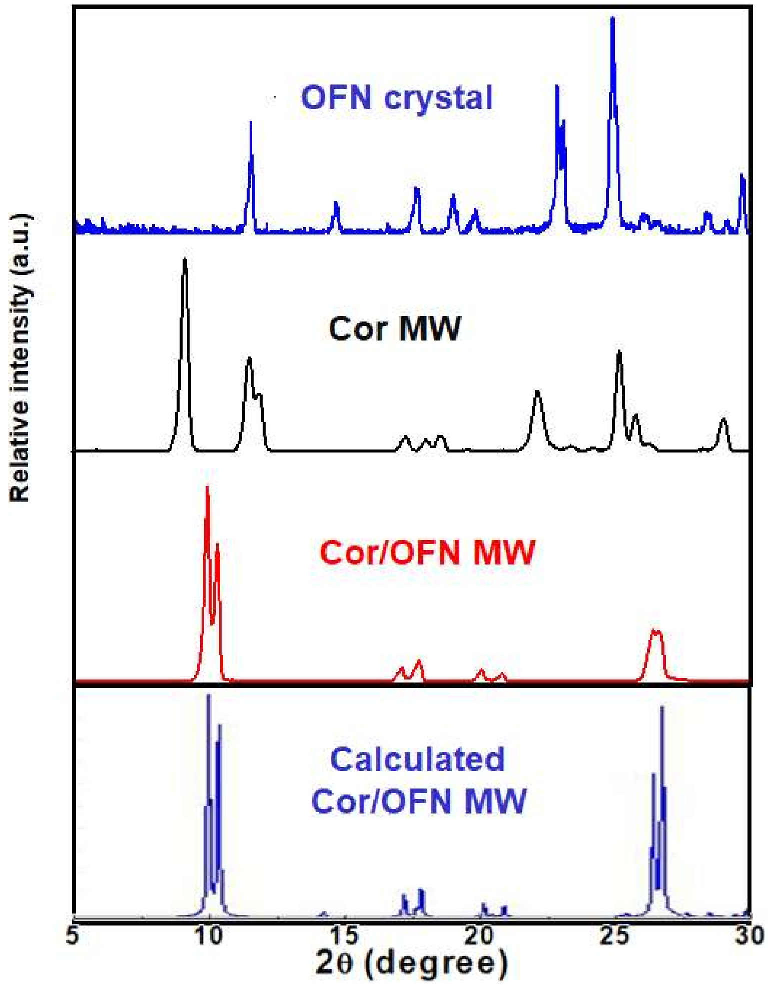

Supplementary Figure 9. Experimental XRD spectra and calculated PXRD for Cor/OFN. Experimental XRD spectra of OFN crystals, Cor MW and Cor/OFN MW and calculated PXRD of Cor/OFN MW.

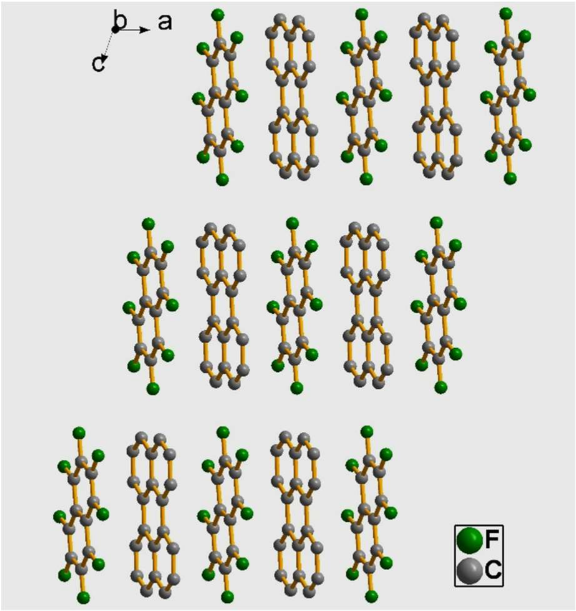

Supplementary Figure 10. Packing structure of Per and OFN. Packing structure of Per and OFN (mole ratio 1:1, CCDC number, 1867077), which shows a mixed stacking mode. Hydrogen atoms are not showed in this structure.

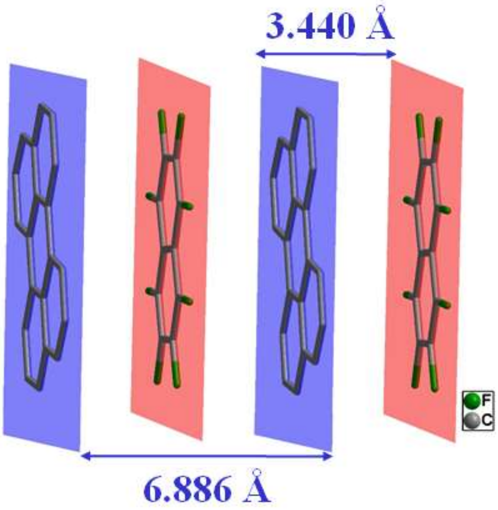

Supplementary Figure 11. The distances between two PAH planes in Per/OFN cocrystals. The distances between two PAH planes in Per/OFN cocrystals obtained from Diamond software. The intercalation of OFN molecules shoved the two adjacent Per-Per molecules and increased the distances to 6.886 Å.

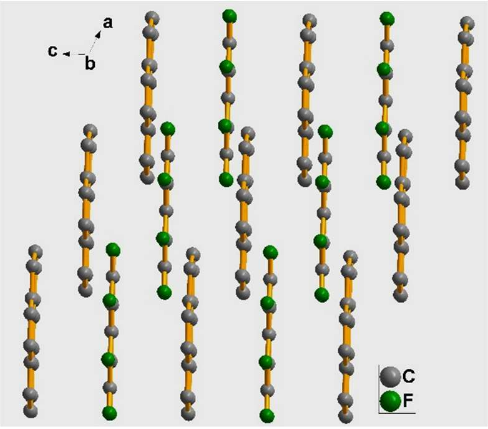

Supplementary Figure 12. Packing structure of Cor and OFN. Packing structure of Cor and OFN (mole ratio 1:1, CCDC number, 1575415), which shows a mixed stacking mode with a mean distance of 3.44 Å between Cor and OFN [1]. Hydrogen atoms are not showed in this Figure.

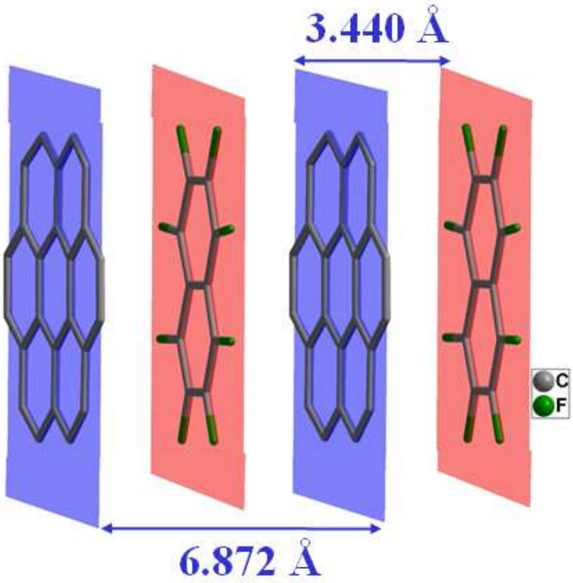

Supplementary Figure 13. The distances between two PAH planes in Cor/OFN cocrystals. The distances between two PAH planes in Cor/OFN cocrystals, obtained from Diamond software. The intercalation of OFN molecules shoved the two adjacent PAH molecules and increased the distances to 6.872 Å.

Supplementary Table 2. Crystallographic data and structure refinement parameters of Ant-OFN co-crystals and Cor-OFN co-crystals.

	Per/OFN	Cor/OFN
T (K)	296	296
Formula	$C_{30}H_{12}F_8$	$C_{34}H_{12}F_8$
Formula weight	524.40	572.44
Crystal system	Triclinic	Monoclinic
Space group	P-1	Cm
Z	1	2
a (Å)	6.886(3)	11.403(11)
b (Å)	8.354(4)	16.99(2)
c (Å)	10.719(5)	6.872(6)
α (deg)	110.720(5)	90
β (deg)	102.299(5)	115.524(10)
γ (deg)	100.177(5)	90
V ($Å^3$)	541.6(4)	1202(2)
ρ calcd (g cm^{-3})	1.608	1.582
λ (Mo Kα) (Å)	0.71073	0.71073
Collected reflns	1885	4288
Unique reflns	1885	2083
Parameters	177	184
R (int)	0	0.0493
R1 [I > 2σ(I)]	0.1087	0.1144
wR2 [I > 2σ(I)]	0.3178	0.3075
GOF	1.123	1.186

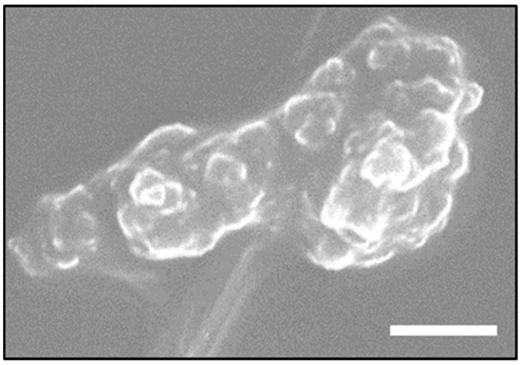

Supplementary Figure 14. SEM image of OFN crystals. SEM image of OFN crystals prepared via the procedure demonstrated in Figure1a. Scale bar is 10 μm.

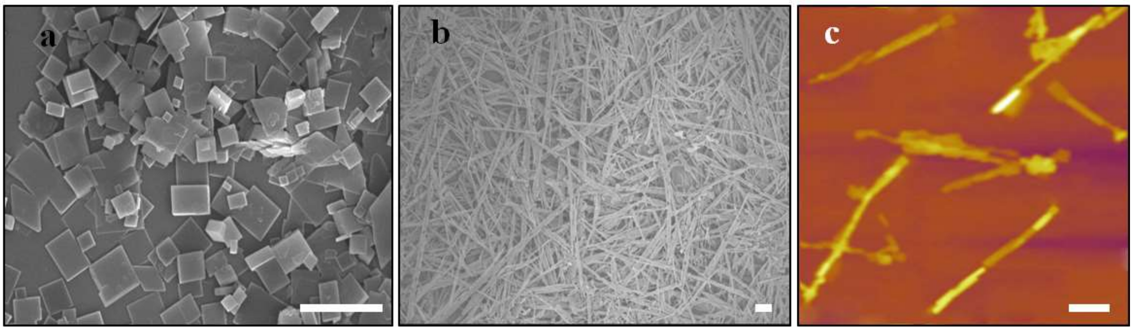

Supplementary Figure 15. SEM and AFM images of Per MS and Per/OFN MW. SEM (a, b) and AFM (c) images of micro sheets of Per MS (a), micro wires of Per/OFN MW (b, c). The scale bars are 5 μm.

Supplementary Table 3. Calculated attachment energies of different crystal facets of Per/OFN co-crystals.

hkl	D_{hkl}/Å	Eatt(Total)/kcal mol^{-1}	%Total facet area
(110)	8.80	-35.54	26.19
(-110)	8.80	-35.54	26.19
(020)	8.50	-36.42	23.35
(11-1)	6.27	-68.54	8.68
(001)	6.20	-69.30	3.45

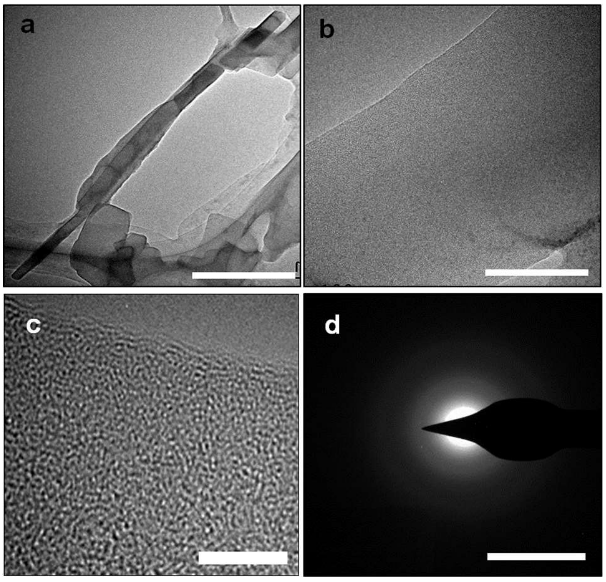

Supplementary Figure 16. TEM images and selected area electron diffraction (SAED) of Per/OFN MW. (a) Low-magnification, high-resolution (b, c) TEM images as well as selected area electron diffraction (SAED) (d) of Per/OFN MW. No diffraction patterns were showed in (d), which were probably attributed to the highly energetic irradiation of electron beams that can destroy crystal structures [7, 8]. The scale bars in (a), (b), (c) and (d) are 5 μm, 500 nm, 5nm and 10/nm, respectively.

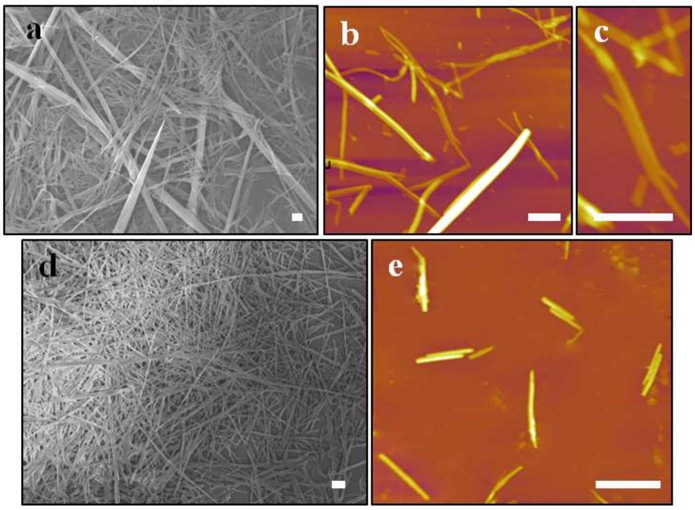

Supplementary Figure 17. SEM and AFM images of Cor MW and Cor /OFN MW. SEM (a, d) and AFM (b, c, e) images of micro wires of Cor MW (a, b and c) and Cor /OFN MW (d, e). The scale bars are 2 μm.

Supplementary Table 4. Calculated attachment energies of different crystal facets of Cor/OFN co-crystal.

hkl	D_{hkl}/Å	Eatt(Total)/kcal mol^{-1}	%Total facet area
(001)	9.57	-14.81	36.94
(01-1)	7.60	-20.77	17.79
(010)	7.51	-19.89	19.77
(100)	6.47	-30.84	11.90
(10-1)	6.30	-31.53	8.69
(1-10)	5.75	-34.37	4.91

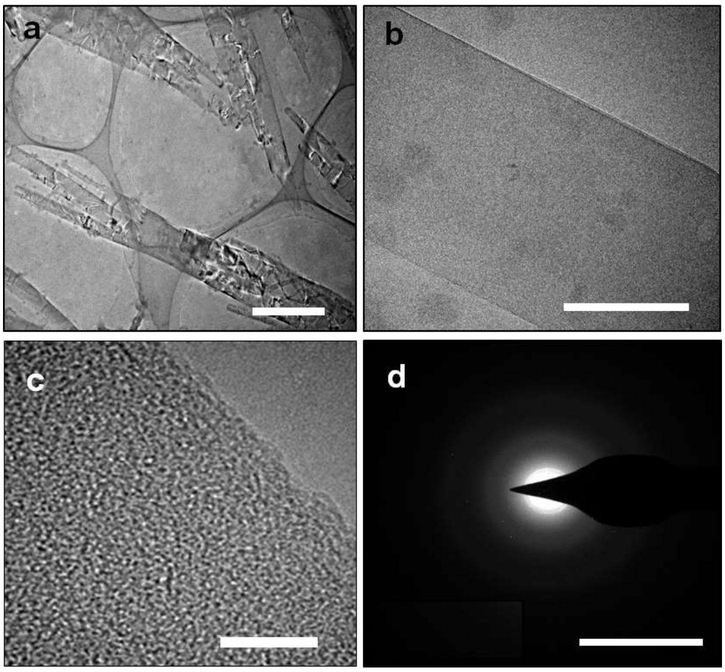

Supplementary Figure 18. TEM images and SAED of Cor/OFN MW. (a) Low-magnification, high-resolution (b, c) TEM images as well as selected area electron diffraction (SAED) (d) of Cor/OFN MW. No diffraction patterns were observed in (d), which were probably attributed to the highly energetic irradiation of electron beams that can destroy crystal structures[7, 8]. The scale bars in (a), (b), (c) and (d) are 500 nm, 100 nm, 5nm and 10/nm, respectively.

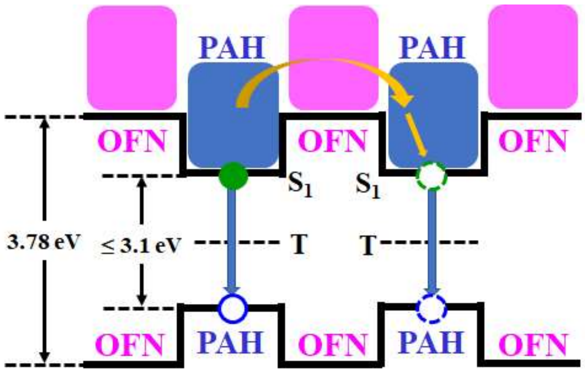

Supplementary Figure 19. Schematic of interactions between OFN and PAHs.[1]

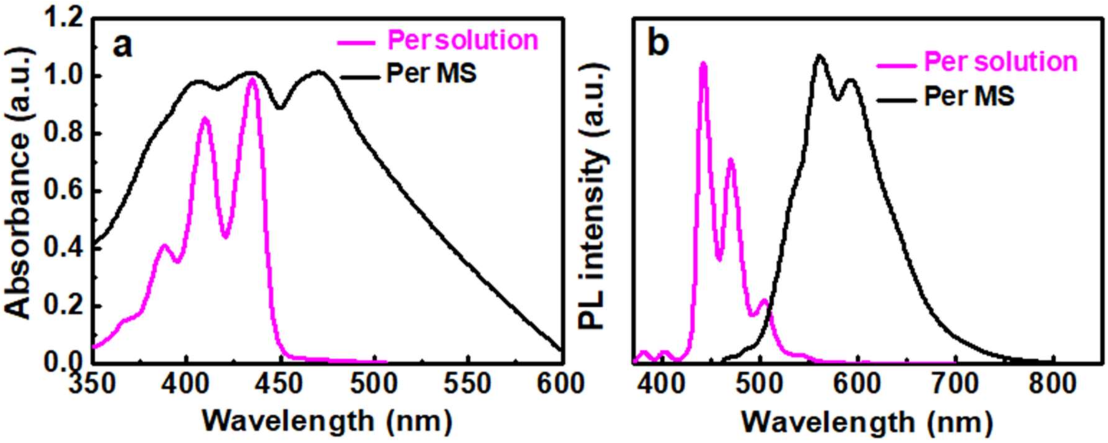

Supplementary Figure 20. Solid UV-vis and PL spectra of THF solution of Per and Per MS powder. Solid UV-vis spectra (a) and PL emission spectra (b) of THF solution of Per (Per solution, purple line) and Per MS powder (black line).

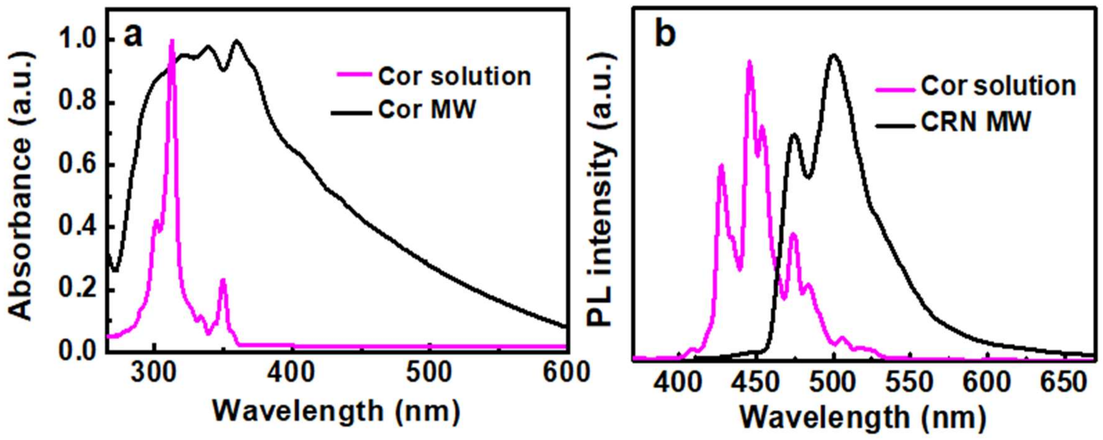

Supplementary Figure 21. UV-vis and PL spectra of THF solution of Cor and Cor MW powder. UV-vis spectra (a) and PL emission spectra (b) of THF solution of Cor (Cor solution, purple line) and Cor MW powder (black line).

The mean fluorescence lifetime was calculated according to the following formula:

$\tau = \Sigma\ B_iT_i^2/\Sigma\ B_iT_i$ (1)

where B_i is the fitting constant, T_i is the lifetime of different decay models. The fitting results were shown in Supplementary Table 5.

Supplementary Table 5. A_i and T_i values of Per MS, Per/OFN MW, Cor MW and Cor/OFN MW.

Sample	T_1 (ns)	B_1 (%)	T_2 (ns)	B_2 (%)
Per MS	6.24	87.1	18.45	12.9
Per/OFN MW	7.38	41.2	17.07	6.9
Cor MW	17.28	59.7	72.62	40.3
Cor/OFN MW	38.65	55.5	189.53	44.5

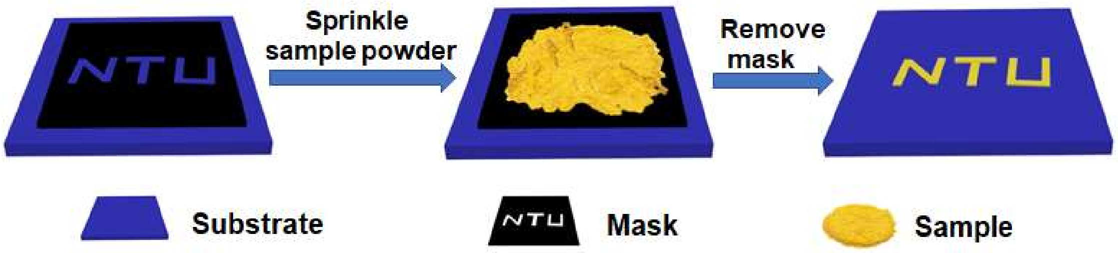

Supplementary Figure 22. The pattern process of "NTU". Firstly, a mask with "NTU" letter shaped holes was putted on the substrate, then the co-crystal powders were sprinkled on the letter area of the mask, followed by dropping a few drops of ethanol. After that, the mask was removed and the "NTU" pattern was presented on the substrate.

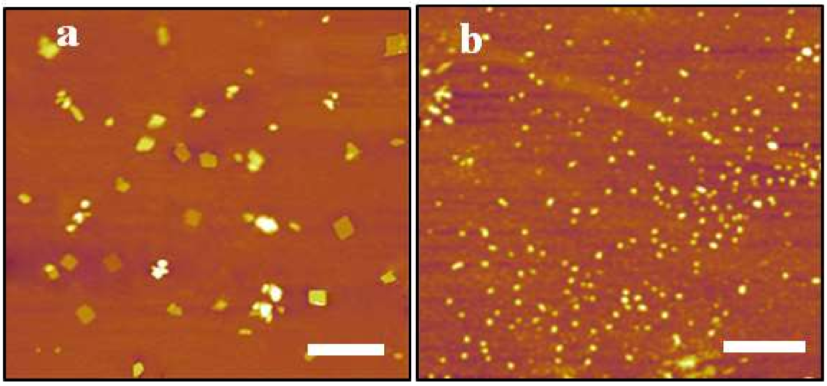

Supplementary Figure 23. AFM images of nano sheets of Per NS (a) and Per /OFN NP (b). The scale bars are 1 μm.

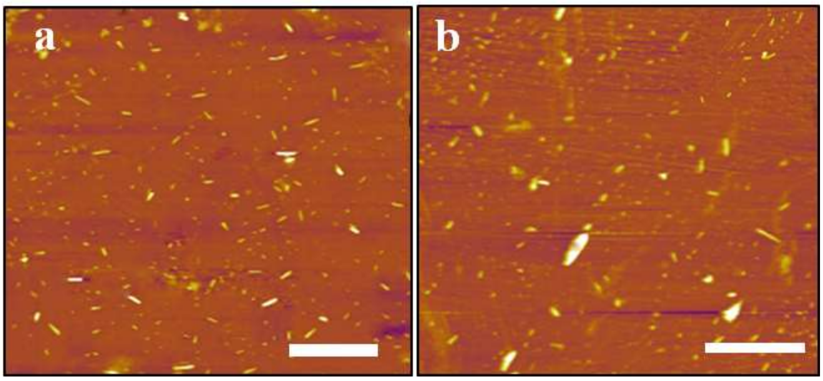

Supplementary Figure 24. AFM images of nano rods of Cor NR (a) and Cor/OFN NR (b). The scale bars are 2 μm.

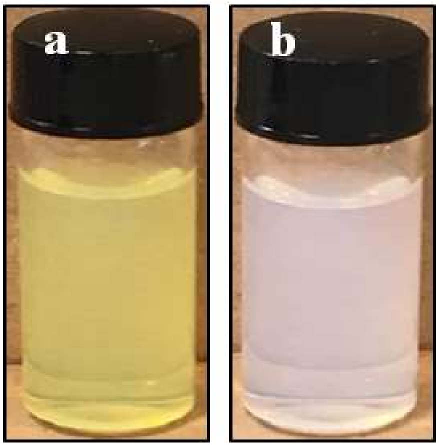

Supplementary Figure 25. Dispersion photos of nano cocrystals after being aged for 6 months. Dispersion photos of Per/OFN NP (a) and Cor/OFN NR (b), respectively, after being aged for 6 months (Concentration of the dispersions, 1 mg mL^{-1}). After being aged for 6 months, the two dispersions were still uniform, and no visualized precipitation appeared.

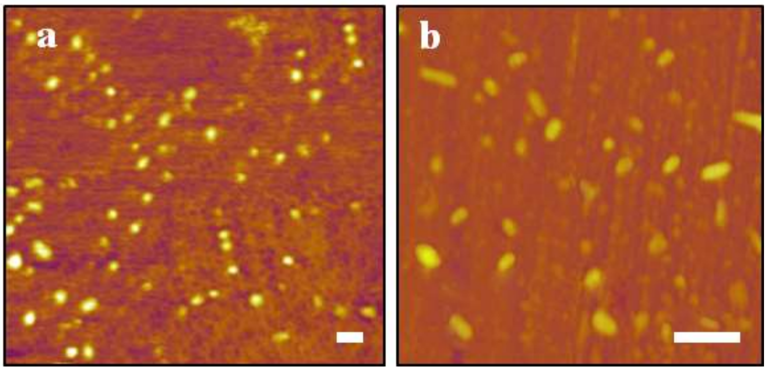

Supplementary Figure 26. AFM images of the nano cocrystals after being aged for 6 months. AFM images of Per/OFN NP (a) and Cor/OFN NR (b), respectively, after being aged for 6 months. After being aged for 6 months, the dimensions of Per/OFN NP and Cor/OFN NR were still smaller than 200 nm, which confirmed the stability of the morphologies of the nano cocrystals. The scale bars are 200 nm.

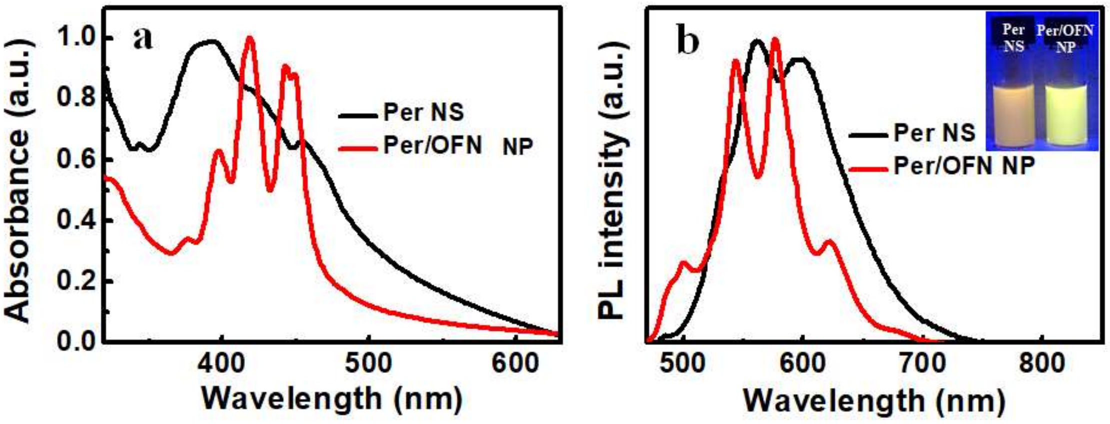

Supplementary Figure 27. UV-vis and PL spectra of the water dispersions for Per NS and Per/OFN NP. UV-vis spectra (a) and PL emission spectra (b) of the water dispersions for Per NS (black line) and Per/OFN NP (red line), respectively. As well as the photographs of the water dispersions (concentration of the dispersions, 1 mg mL^{-1}) under irradiation of 365 nm laser (inset in b). Just like the micro cocrystals, being co-stacked with electron-deficient OFN molecules, the absorption and emission spectra of the Per/OFN NP also blue-shifted evidently.

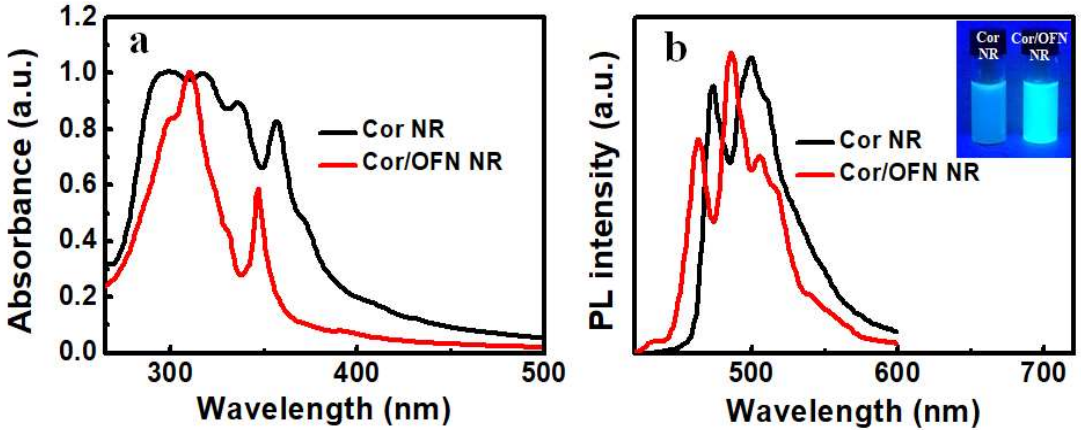

Supplementary Figure 28. UV-vis and PL spectra of the water dispersions for Cor NR and Cor/OFN NR. UV-vis spectra (a) and PL emission spectra (b) of the water dispersions for Cor NR (black line) and Cor/OFN NR (red line), respectively. As well as the photographs of the water dispersions (concentration of the dispersions, 1 mg mL^{-1}) under irradiation of 365 nm laser (inset in b). After being doped by electron-deficient OFN molecules, the absorption and emission spectra of the Per/OFN NP blue-shifted evidently.

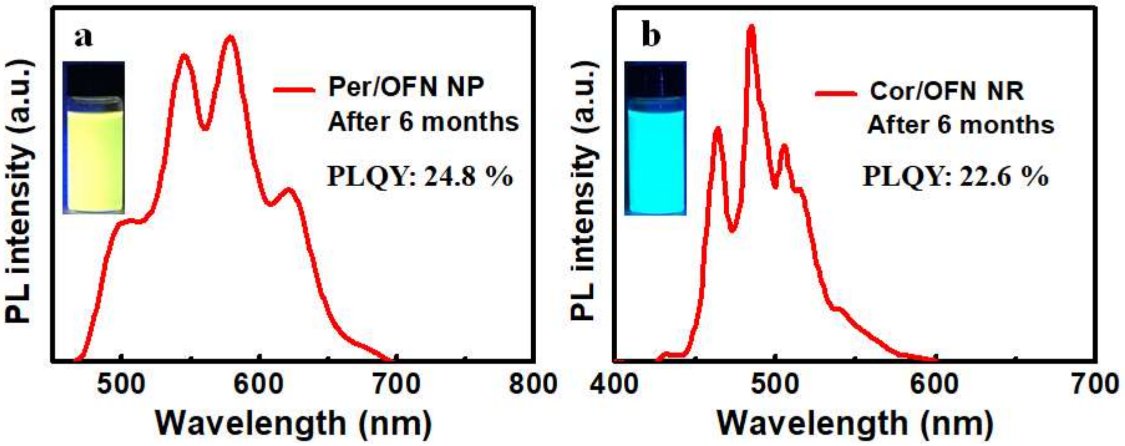

Supplementary Figure 29. PL spectra of the water dispersions for the nano cocrystals after 6 months placement. PL emission spectra of the water dispersions for Per/OFN NP (a) and Cor/OFN NR (b), respectively, after 6 months placement. As well as the photographs of the water dispersions (concentration of the dispersions, 1 mg mL^{-1}) under irradiation of 365 nm laser after 6 months (insets). The PL spectra of the two nano cocrystals presented the same location and shape as that of the freshly prepared ones demonstrated in Supplementary Figure 26(b) and 27(b). The PLQYs of aged Per/OFN NP and Cor/OFN NR were 24.8% and 22.6 %, which were close to that of the freshly prepared ones, further confirmed the stability of the dispersions.

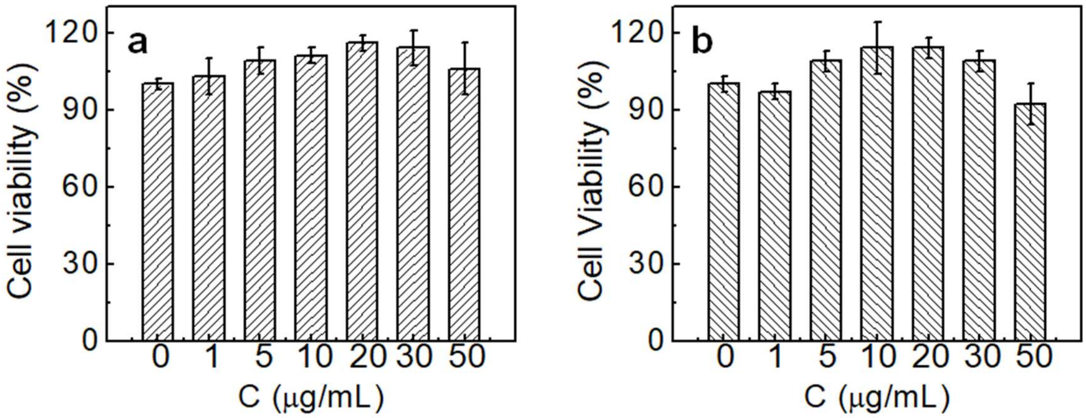

Supplementary Figure 30. Cytotoxicity tests. Cytotoxicity tests on MCF-7 cells at different particle concentrations (0, 1, 5, 10, 20, 30, 50 μg mL^{-1}) for Per/OFN NP (a) and Cor/OFN NR (b) after 24 h incubation. The cell viability was assessed via MTT assay. The values presented are the mean ± SD (n = 3).

Supplementary Reference

1. Ye, H. et al. Molecular-Barrier-Enhanced Aromatic Fluorophores in Cocrystals with Unity Quantum Efficiency. *Angew. Chem. Int. Ed.* **57**, 1928-1932 (2018).
2. Ding, D. et al. Bioprobes Based on AIE Fluorogens. *Acc. Chem. Res.* **46**, 2441-2453 (2013).
3. Borisov, S. M. et al. Optical biosensors. *Chem. Rev.* **108**, 423-461 (2008).
4. Alexandridis, P. et al. Micellization of Poly(ethylene oxide)-Poly(propylene oxide)-Poly(ethylene oxide) Triblock Copolymers in Aqueous Solutions: Thermodynamics of Copolymer Association. *Macromolecules* **27**, 2414-2425 (1994).
5. Hanson, G. R. et al. Halogen Bonding between an Isoindoline Nitroxide and 1,4-Diiodotetrafluorobenzene: New Tools and Tectons for Self-Assembling Organic Spin Systems. *Chem. - Eur. J.* **15**, 4156-4164 (2009).
6. Kenji, Y. et al. A Study of Electronic Structure of 1,2,4,5-Tetracyanobenzene Anion Radical by Resonance Raman Effect. *Chem. Soc. Jpn.* **53**, 1949-1955 (1980).
7. Huang, Y. et al. Temperature-Dependent Multidimensional Self-Assembly of Polyphenylene-Based “Rod-Coil” Graft Polymers. *J. Am. Chem. Soc.* **137**, 11602-11605 (2015).
8. Egerton, R. F. et al. Radiation damage in the TEM and SEM. *Micron* **35**, 399-409 (2004).
